# SDF1–CXCR4 signaling contributes to persistent pain and hypersensitivity via regulating excitability of primary nociceptive neurons: involvement of ERK-dependent Nav1.8 up-regulation

**DOI:** 10.1186/s12974-015-0441-2

**Published:** 2015-11-24

**Authors:** Fei Yang, Wei Sun, Yan Yang, Yan Wang, Chun-Li Li, Han Fu, Xiao-Liang Wang, Fan Yang, Ting He, Jun Chen

**Affiliations:** Institute for Biomedical Sciences of Pain, Tangdu Hospital, The Fourth Military Medical University, #569 Xinsi Road, Baqiao, Xi’an, 710038 People’s Republic of China; Key Laboratory of Brain Stress and Behavior, PLA, Xi’an, 710038 People’s Republic of China; Beijing Institute for Brain Disorders, Beijing, 100069 People’s Republic of China

**Keywords:** Inflammatory pain, Chemokines, SDF1, CXCR4, Nav1.8, Dorsal root ganglion, ERK signaling

## Abstract

**Background:**

Pain is one critical hallmark of inflammatory responses. A large number of studies have demonstrated that stromal cell-derived factor 1 (SDF1, also named as CXCL12) and its cognate receptor C-X-C chemokine receptor type 4 (CXCR4) play an important role in immune reaction and inflammatory processes. However, whether and how SDF1–CXCR4 signaling is involved in inflammatory pain remains unclear.

**Methods:**

Under the intraplantar (i.pl.) bee venom (BV) injection-induced persistent inflammatory pain state, the changes of SDF1 and CXCR4 expression and cellular localization in the rat dorsal root ganglion (DRG) were detected by immunofluorescent staining. The role of SDF1 and CXCR4 in the hyperexcitability of primary nociceptor neurons was assessed by electrophysiological recording. Western blot analysis was used to quantify the DRG Nav1.8 and phosphorylation of ERK (pERK) expression. Behavioral tests were conducted to evaluate the roles of CXCR4 as well as extracellular signal-regulated kinase (ERK) and Nav1.8 in the BV-induced persistent pain and hypersensitivity.

**Results:**

We showed that both SDF1 and CXCR4 were dramatically up-regulated in the DRG in i.pl. BV-induced inflammatory pain model. Double immunofluorescent staining showed that CXCR4 was localized in all sizes (large, medium, and small) of DRG neuronal soma, while SDF1 was exclusively expressed in satellite glial cells (SGCs). Electrophysiological recording showed that bath application with AMD3100, a potent and selective CXCR4 inhibitor, could reverse the hyperexcitability of medium- and small-sized DRG neurons harvested from rats following i.pl. BV injection. Furthermore, we demonstrated that the BV-induced ERK activation and Nav1.8 up-regulation in the DRG could be blocked by pre-antagonism against CXCR4 in the periphery with AMD3100 as well as by blockade of ERK activation by intrathecal (i.t.) or intraplantar (i.pl.) U0126. At behavioral level, the BV-induced persistent spontaneous pain as well as primary mechanical and thermal hypersensitivity could also be significantly suppressed by blocking CXCR4 and Nav1.8 in the periphery as well as by inhibition of ERK activation at the DRG level.

**Conclusions:**

The present results suggest that peripheral inflammatory pain state can trigger over release of SDF1 from the activated SGCs in the DRG by which SGC-neuronal cross-talk is mediated by SDF1–CXCR4 coupling that result in subsequent ERK-dependent Nav1.8 up-regulation, leading to hyperexcitability of tonic type of the primary nociceptor cells and development and maintenance of persistent spontaneous pain and hypersensitivity.

## Background

Persistent pain that results from inflammation is a major public health problem worldwide. An increasing number of evidence has demonstrated that the up-regulation of inflammatory mediators, including cytokines and chemokines, is involved in the generation of neuronal hyperexcitability and thus contributes to inflammatory pain and hyperalgesia [1–6].

Chemokines could be sorted into four families: C family, CC family, CXC family, and CX3C family [7]. Stromal cell-derived factor 1 (SDF1, also named as CXCL12), as a member of the CXC family, is constitutively expressed in various kinds of cells in the peripheral and central nervous system [8, 9]. Through activating the C-X-C chemokine receptor type 4 (CXCR4), a seven transmembrane G-protein-coupled receptor, SDF1 exerts multiple biological functions [9]. In addition to the well-established role in the immune system and inflammatory reaction, emerging data have shown that SDF1/CXCR4 system is also involved in neurogenesis, neuronal migration, and neuronal differentiation during the development of the nervous system [10–14]. Recently, the pro-algesic effects of SDF1/CXCR4 have also been implicated in several pain models. Immunohistochemical studies found that the expression level of SDF1 and CXCR4 was changed in the dorsal root ganglion (DRG) cells in the unilateral sciatic nerve injury (CCI)-induced pain model and the up-regulation of CXCR4 lasted at least for 2 weeks [15]. In the spinal cord injury-induced central neuropathic pain model, Knerlich-Lukoschus and colleagues demonstrated that SDF1 and CXCR4 expression was continuously increased from 2 to 42 days at the spinal cord level [16]. Moreover, by mapping the cellular and subcellular localization of SDF1 and CXCR4, Reaux-Le and colleagues detailedly reported that SDF1/CXCR4 system was closely related to the nociceptive pathway, especially in the primary nociceptive neurons, and they also found that activating the CXCR4 by intrathecal (i.t.) SDF1 injection could induce mechanical allodynia for 3 days, which could be prevented by the CXCR4-neutralizing antibody [8]. However, to date, the underlying mechanisms of SDF1/CXCR4 involved in the chronic and persistent pain remain unclear.

Recently, in vitro electrophysiological experiments have verified that SDF1 could directly modulate the excitability and firing pattern of neuronal cells through CXCR4 [17–21]. As for neurons, voltage-gated sodium channels (VGSCs) are the key mediators in cellular excitability and are essential for the generation and propagation of action potentials (AP) [22, 23]. Among all the VGSCs, tetrodotoxin-resistant (TTX-R) sodium channel Nav1.8 and/or Nav1.9 mostly contributes to the enhanced excitability and produces majority of current during AP upstroke [24]. Accumulating data showed that peripheral inflammation and nerve injury could induce up-regulation of Nav1.8 in the medium- and small-sized DRG neurons [25–29]. Furthermore, several studies observed that inflammatory mediators (including TNF-α, CCL2, and CXCL1) could directly induce Nav1.8 up-regulation in the DRG and thus excite the primary nociceptive neurons [26, 30, 31]. Taking all these data into account, we hypothesized that the above modulating effect of the SDF1/CXCR4 system on the neuronal excitability was in part due to the regulation of Nav1.8 which in turn contributes to the generation of pain.

Subcutaneous intraplantar (i.pl.) injection of bee venom (BV) is a frequently used inflammatory pain model which is suitable for exploring the pathophysiological mechanism of persistent pain and inflammation [32–34]. Thus, in the present study, we firstly examined the expression of CXCR4 and SDF1 in DRG cells under inflammatory pain condition induced by i.pl. BV injection. Then, we investigated the role of CXCR4 in the neuronal excitability and Nav1.8 modulation by applying the selective inhibitor of CXCR4, AMD3100. Finally, we investigated whether blocking CXCR4 could alleviate the inflammatory pain-related behaviors.

## Methods

### Animals

The experiments were performed on male Sprague–Dawley rats weighing from 180 to 220 g (purchased from the Laboratory Animal Center of Fourth Military Medical University, FMMU, Xi’an, People’s Republic of China). The animals were housed in plastic boxes with access to water and food ad libitum and maintained on a 12-h light/dark cycle (with the lights on at 8:00 a.m. to 8:00 p.m.) at room temperature (22–26 °C). Behavioral evaluations were carried out between 9:00 and 18:30. The rats were acclimated to test boxes for at least 30 min each day for 5 days before testing. The present experiment protocols were approved by the Institutional Animal Care and Use Committee of FMMU, and were in accordance with the National Institutes of Health Guide for the Care and Use of Laboratory Animals (NIH Publications No. 80-23) revised 1996. International Association for the Study of Pain (IASP)’s ethical guidelines for pain research in conscious animals was followed. The number of animals used and their suffering were greatly minimized.

### Behavioral testing

#### Persistent spontaneous nociceptive behavior

The method to estimate persistent spontaneous nociception (PSN) was based on our previously reports [34]. A 30 × 30 × 30 cm transparent Plexiglas box was placed on a supporting frame of 30 cm high above the experimental table. The rat was placed in the test box for at least 30 min before administration of any chemical agents. After the acclimation period, an i.pl. injection of BV was made into the center of the plantar surface of one hind paw with slight restraint. BV-induced persistent spontaneous nociception was expressed as the number of paw finches occurring at each 5-min interval for 1 h following i.pl. BV injection.

#### Mechanical pain sensitivity

For examination of mechanical pain sensitivity, the mechanical stimuli were applied by using ascending graded individual von Frey monofilaments with bending forces of 0.8, 2.0, 4.0, 6.0, 8.0, 10.0, 12.0, 14.0, 16.0, 18.0, 20.0, 25.0, 30.0, 45.0, and 60 g. The rats were placed on a metal mesh floor covered with plastic box, and von Frey filaments were applied from underneath the metal mesh floor to the plantar area of the bilateral hind paws 1 h before and 2 h after i.pl. BV injection. Each von Frey filament was applied ten times (once every several seconds) in order to induce the withdrawal reflex. The bending force value of the von Frey filament that caused an appropriate 50 % occurrence of paw withdrawal was expressed as the paw withdrawal mechanical threshold (PWMT, g). For details, see [32, 34].

#### Thermal pain sensitivity

The thermal sensitivity was determined by measuring the withdrawal latency of the hind paws in response to radiant heat [34]. Rats were placed in a plastic chamber on the surface of a 2-mm thick glass plate and the sensitivity to heat stimuli by a TC-1 radiant heat stimulator (new generation of RTY-3 made in Xi’an Bobang Technologies of Chemical Industry Co. Ltd., China, 10 V) at 30 min before and 3 h after i.pl. BV treatment was measured. The heat stimuli were applied to both the injection site and the corresponding area of the contralateral paw, and the latency was determined as the duration from the beginning of heat stimuli to the occurrence of a marked withdrawal reflex. Five stimuli were repeated for each site, and the latter three values were averaged as mean paw withdrawal thermal latency (PWTL, s). A maximal cutoff of 30 s was used to avoid excessive tissue injury. The inter-stimulus interval for each heat test was more than 15 min at the same region and 10 min at the different paws.

### Drug

A volume of 50 μl BV solution (4 μg/μl, Floret Ltd. and its partner company New Techniques Laboratory Ltd., Tbilisi, Georgia, dissolved in 0.9 % sterile saline) was used during the whole experiment. To evaluate the role of CXCR4 in the PSN, thermal, and mechanical pain sensitivity, AMD3100 (10, 100, or 200 μg/20 μl, Sigma) or vehicle was administered through subcutaneous i.pl. injection 10 min prior to BV treatment. We chose this route of delivery to exclude the spinal roles of CXCR4 in the BV-induced pathological pain processing. To evaluate the role of extracellular signal-regulated kinase (ERK), an isoform of mitogen-activated protein kinase (MAPK), U0126 (Sigma, 10 μg dissolved in 10 μl DMSO), which was decided according to our previous study [35, 36], was administered through intrathecal (i.t.) or intraplantar (i.pl.) injection 10 min prior to BV treatment. Considering the effective duration and peak time, A-803467 (Abcam, 500 μg/50 μl dissolved in DMSO), a selective Nav1.8 blocker, was administered through subcutaneous injection 10 min prior to or 90 min post-BV treatment [37, 38].

### Implantation of intrathecal catheters and administration of drug

Intrathecal catheter implantation was performed according to the method described previously [39]. Briefly, under sodium pentobarbital anesthesia (40 mg/kg, i.p.), a sterile polyethylene (PE-10) tube filled with 0.9 % sterile saline was inserted into the L5/L6 intervertebral space, and the tip of the tube was placed at the spinal lumbar enlargement level. The tube was fixed by suturing into the superficial muscle. Then, a tunnel under the skin was made and the tube was pulled out of another skin incision at the neck area where the tube was fixed on the skin. The outer end of the catheter was sealed by melting. The rats were allowed to recover for 3 days in individual cages, and only those without motor disturbance and other neurological deficits were included for further experiments. Drugs or vehicle were administered in volumes of 10 μl by microinjection syringe followed by a flush of 10 μl saline to ensure drugs were delivered into the subarachnoid space. After drug injection, the outer end of the catheter was sealed again by heat melting.

### Immunohistochemistry

The rats were anesthetized with 1 % pentobarbital sodium (50 mg/kg, i.p.), then perfused with physiological saline, followed by 4 % paraformaldehyde in 0.1 M PB solution. After perfusion, the L4–6 DRGs were removed and postfixed in the same 4 % fixative overnight at 4 °C and cryoprotected by immersion in 30 % sucrose in 0.1 M PB at 4 °C till it is sunk on the bottom of the container. Transverse frozen sections (15 μm thick) were cut on CM1900 freezing microtome (Leica, Germany). Sections were blocked with 10 % goat serum in phosphate buffered saline (PBS) for 2 h at room temperature and incubated with primary antibody at 4 °C overnight. The primary antibodies used are listed in Table 1. After three washes with PBS, the sections were incubated with secondary antibodies for 2–3 h at room temperature. For double immunofluorescence, sections were incubated with a mixture of primary antibodies overnight at 4 °C, followed by a mixture of FITC-conjugated and Cy3-conjugated secondary antibodies. For IB4 labeling, the Alexafluor 594-conjugated isolectin IB4 from *Griffonia simplicifolia* was used for bioaffinity labeling. The images were examined under a laser scan confocal fluorescent microscope (Olympus FV1000, Japan).

**Table 1:** Antibodies for immunofluorescence and Western blotting

Antibodies	Host	Vendor
SDF-1	Goat	Santa Cruz
CXCR4	Goat	Santa Cruz
IB4	–	Life Technologies
Substance P	Rabbit	Chemicon
TRPV1	Rabbit	Alomone
pERK	Mouse	Santa Cruz
Nav1.8	Rabbit	Alomone
GFAP	Mouse	Millipore

### Western blotting

Rats were sacrificed by decapitation after behavioral testing, and the L4–6 DRGs ipsilateral to BV injection were obtained and homogenized in a RIPA lysis buffer containing protease inhibitors (Applygen Technologies Inc., China). Protein concentrations of the lysate were determined using a BCA Protein Assay kit (Thermo Scientific, Rockford, IL, USA). Protein samples were heated for 10 min at 95 °C with SDS-PAGE sample buffer, and equal amounts of protein were then separated by 10 % separation gels. The resolved proteins were subsequently transferred to nitrocellulose membranes (Bio-Rad, Hercules, CA, USA) followed by the incubation with 5 % nonfat milk (Bio-rad, CA, USA) in PBS with 0.05 % Tween 20 (PBST) for at least 30 min at room temperature. Then, the membranes were incubated with primary antibodies at 4 °C overnight. The primary antibodies used are listed in Table 1. After washing three times in PBST, the membranes were incubated for 2 h at room temperature with an HRP-conjugated secondary antibody (1:2000, Bio-Rad). The membranes were visualized with enhanced chemiluminescencce solution (Alpha Innotech Corp.), and the signals were captured with FluorChem FC2 (Alpha Innotech Corp.). The density of specific bands was measured with a computer-assisted imaging analysis system (Bio-Rad, CA, USA) and normalized to β-tubulin intensity.

### Intact DRG preparation and electrophysiological recording

Rats were anesthetized with 1 % pentobarbital sodium (50 mg/kg, i.p.); L4–6 DRGs were harvested for electrophysiological recording 2 h after BV injection when the maximal effect was seen for pain hypersensitivity. The whole DRGs were placed into an artificial cerebrospinal fluid maintained at 4 °C (ACSF, contained in mM: 124 NaCl, 2.5 KCl, 1.2 NaH_2_PO_4_, 1.0 MgCl_2_, 2.0 CaCl_2_, 25 NaHCO_3_, and 10 glucose). Then, the DRGs were incubated with digestive solution containing 0.4 mg/ml trypsin (Sigma) and 1.0 mg/ml type-A collagenase (Sigma) for 40 min at 37 °C. After digestion, all DRGs were incubated in carbogen gas-bubbled ACSF at room temperature (25–28 °C) for at least 2 h.

For electrophysiological recording, the ganglion was transferred to the recording chamber which was perfused with carbogen gas-bubbled ACSF at room temperature. A small mesh anchor was used to keep the ganglion stabilized. The neurons in DRG were visualized with ×40 water-immersion objective attached to a BX51WI microscope (BX51WI, Olympus, Japan) equipped with infrared-differential interference contrast optics. All recordings were made with EPC10 amplifier and Pulse software (HEKA Elektronik, Germany). Current-clamp recordings were made to evoke action potentials and measure the changes of membrane potential using the whole-cell patch-clamp technique. The patch pipettes were fabricated with P-97 Puller (Narishige, Japan) and had resistances of 4–7 MΩ with internal solution before seal formation. The internal pipette solution contained the following (in mM): 140 KCl, 2 MgCl_2_, 10 HEPES, and 2 Mg-ATP (pH 7.4, adjusted by KOH). Osmolarity was adjusted to 290–300 mOsm by sucrose. All junction potentials were corrected online by adjusting the pipette offset. After GΩ-seal whole-cell formed under voltage-clamp holding at−60 mV, capacitance transient was canceled and serious resistance was compensated (80–90 %) digitally. Neurons were selected for further study if they had a resting membrane potential more negative than −50 mV and exhibited an overshooting action potential.

### Statistical analysis

All data were expressed as mean ± SEM. Differences in changes of values of each group were tested using *t* tests and one-way ANOVA, followed by individual post hoc comparisons (Tukey or Bonferroni test). A level of *P* < 0.05 was accepted as significant.

## Results

### Localization of CXCR4 within primary nociceptor neurons

**Fig. 1:**
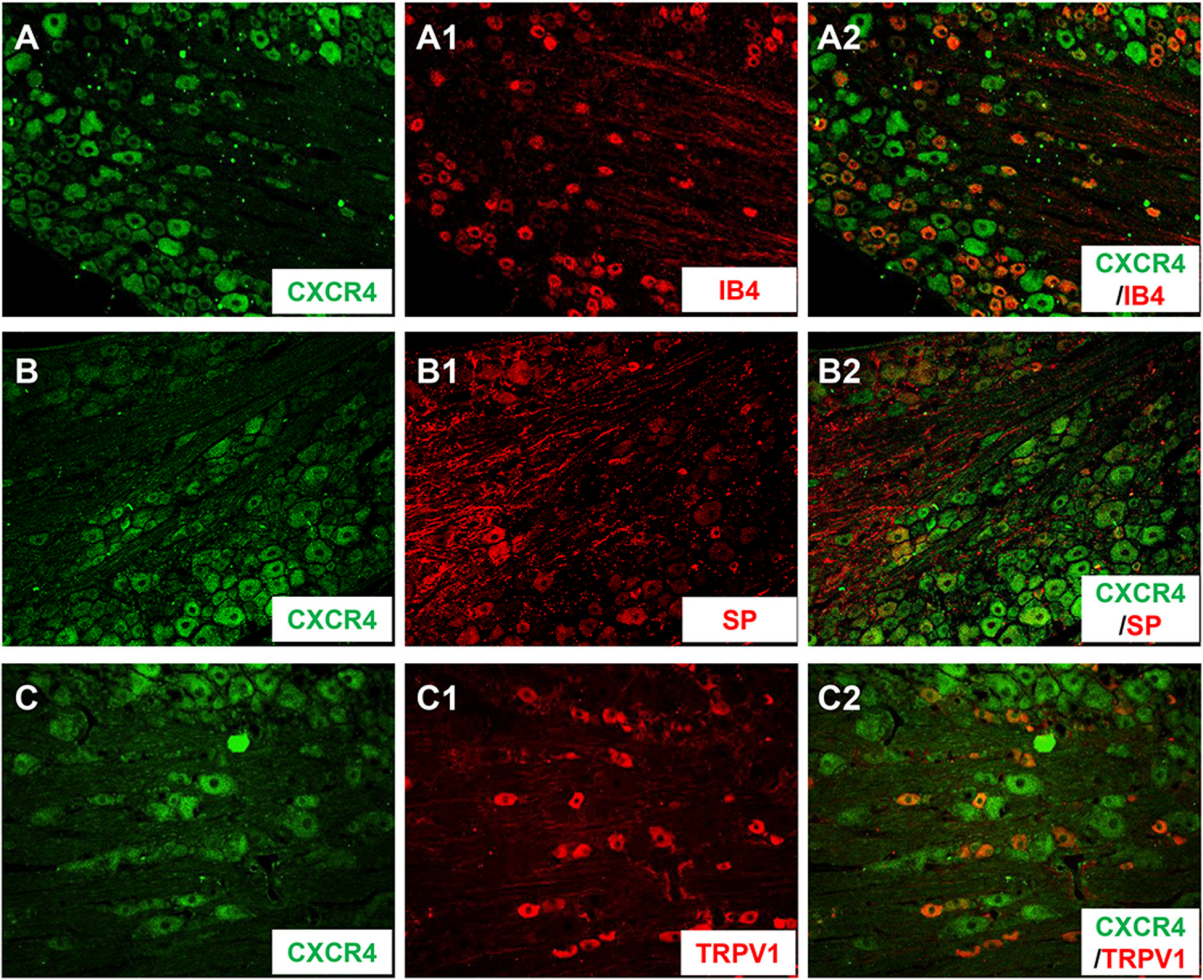
Localization of CXCR4 in rat primary nociceptive neurons. Immunofluorescence micrographs show the double-staining of CXCR4 (**a**–**c**) with IB4 (**a1**), substance P (SP, **b1**), and TRPV1 (**c1**). Showing merged images from **a1** and **a2**, **b1** and **b2**, **c1** and **c2**. Note that CXCR4 was co-localized with IB4, SP, and TRPV1 in subpopulations of DRG neurons (**a2**–**c2**)

Under naïve state, CXCR4 was localized in almost all non-peptidergic IB4-positive cells (Fig. 1a–a2); however, CXCR4 was only seen in a few number of peptidergic SP-positive cells (Fig. 1b–b2). Moreover, CXCR4 was also localized in most of TRPV1-positive neurons (Fig. 1c–c2). CXCR4 was also seen in NF-200 positive neurons (data not shown).

### Up-regulation of SDF1 and CXCR4 in the dorsal root ganglia induced by peripheral inflammatory pain state

**Fig. 2:**
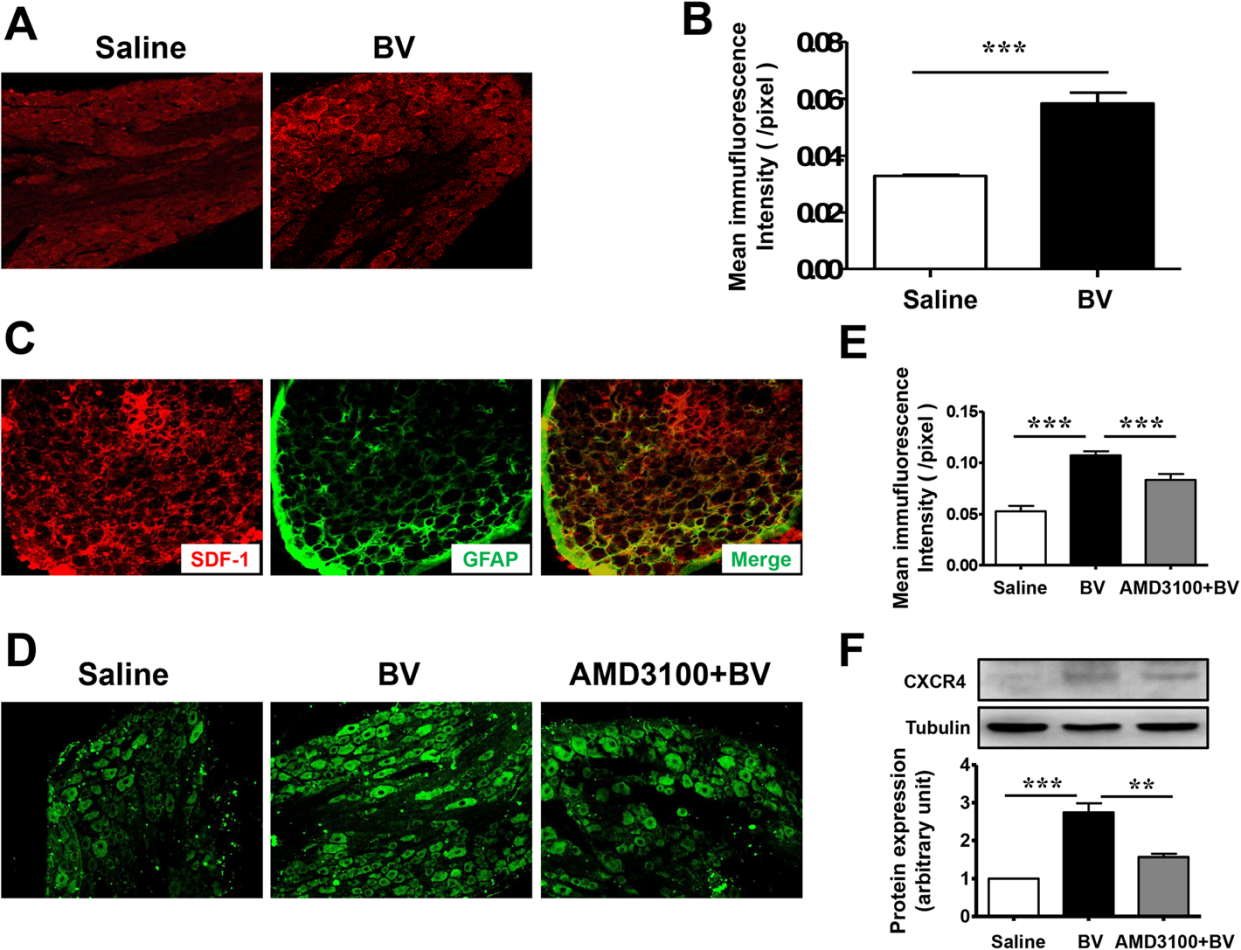
Intraplantar BV injection induces up-regulation of SDF1 and CXCR4 in the rat lumbar DRG. **a** Representative immunofluorescence photomicrographs of SDF1 in lumbar DRG from saline-treated rats and BV-treated rats. **b** Quantification of the mean immunofluorescent intensity of SDF1 showing an increase in SDF1 expression following intraplantar BV injection (*n* = 5/group, ***P < 0.001). **c** Immunofluorescent photomicrographs of double-staining of the SDF1 (*red*) with GFAP (*green*), a marker for satellite glial cell. **d** Representative immunofluorescent photomicrographs of CXCR4 in the lumbar DRG from saline-treated rats, BV-treated rats, and rats that received AMD3100 10 min before BV injection. **e** Quantification of the mean immunofluorescent intensity of CXCR4 showing an increase in CXCR4 expression following intraplantar BV injection and a reduction of CXCR4 in rats that received AMD3100 10 min before injection (*n* = 5/group, ***P < 0.001). **f** Western blot showing the expression of CXCR4 in lumbar DRG from saline-treated rats, BV-treated rats, and rats that received AMD3100 10 min before BV injection. Representative bands are shown on the top, and data summary is shown on the bottom. (*n* = 3/group, **P < 0.01, ***P < 0.001)

Under peripheral inflammatory pain state induced by i.pl. BV injection, the SDF1-like immunoreactivity was significantly increased in the DRG when compared with the saline control in which less SDF1 immunoreactivity could be seen (Fig. 2a, b). Because the BV-induced increase in SDF1-like immunoreactivity was not seen in the DRG neuronal profiles, but was seen in intercellular space, we performed a double immunofluorescent labeling for SDF1 and glial fibrillary acidic protein (GFAP), a marker for satellite glial cells of the DRG. As seen in Fig. 2c, SDF1 was localized in almost all GFAP-positive profiles in the DRG (Fig. 2c).

We next sought to examine the level of CXCR4 expression in the DRG under BV injection-induced inflammatory pain state. As shown in Fig. 2d–f, the expression level of CXCR4 was also significantly increased in the DRG neurons relative to saline control demonstrated by both immunofluorescent staining and Western blotting analysis. However, the up-regulation of CXCR4 induced by i.pl. BV injection could be significantly prevented by pre-treatment with subcutaneous AMD3100, a selective CXCR4 inhibitor, suggesting that the BV-induced overexpression of CXCR4 in the DRG cells may be partially mediated by peripheral SDF1–CXCR4 signaling in the skin through trafficking to the peripheral terminals. The BV-induced increases in both SDF1 and CXCR4 were restricted to the ipsilateral side to the BV injection, but with the level of them being unchanged in the contralateral side (not shown).

### Inhibition of the BV-induced hyperexcitable state of primary nociceptor neurons by AMD3100, a selective antagonist of CXCR4

Similar to our previous reports [28, 29, 40, 41], the primary nociceptor neurons could be persistently activated by peripheral inflammatory pain state induced by i.pl. injection of both bee venom and complete Freund’s adjuvant (CFA) through up-regulation of both Nav1.8 and Nav1.9 of VGSC in the DRG cells. However, only the tonic, but not the phasic, type of primary nociceptor neurons was involved in this processing [41]. The phasic type of the DRG neurons is characterized by producing only one AP following repeated electrical stimulation; however, the tonic type is featured by producing many APs following the same electrical stimulation [41].

**Fig. 3:**
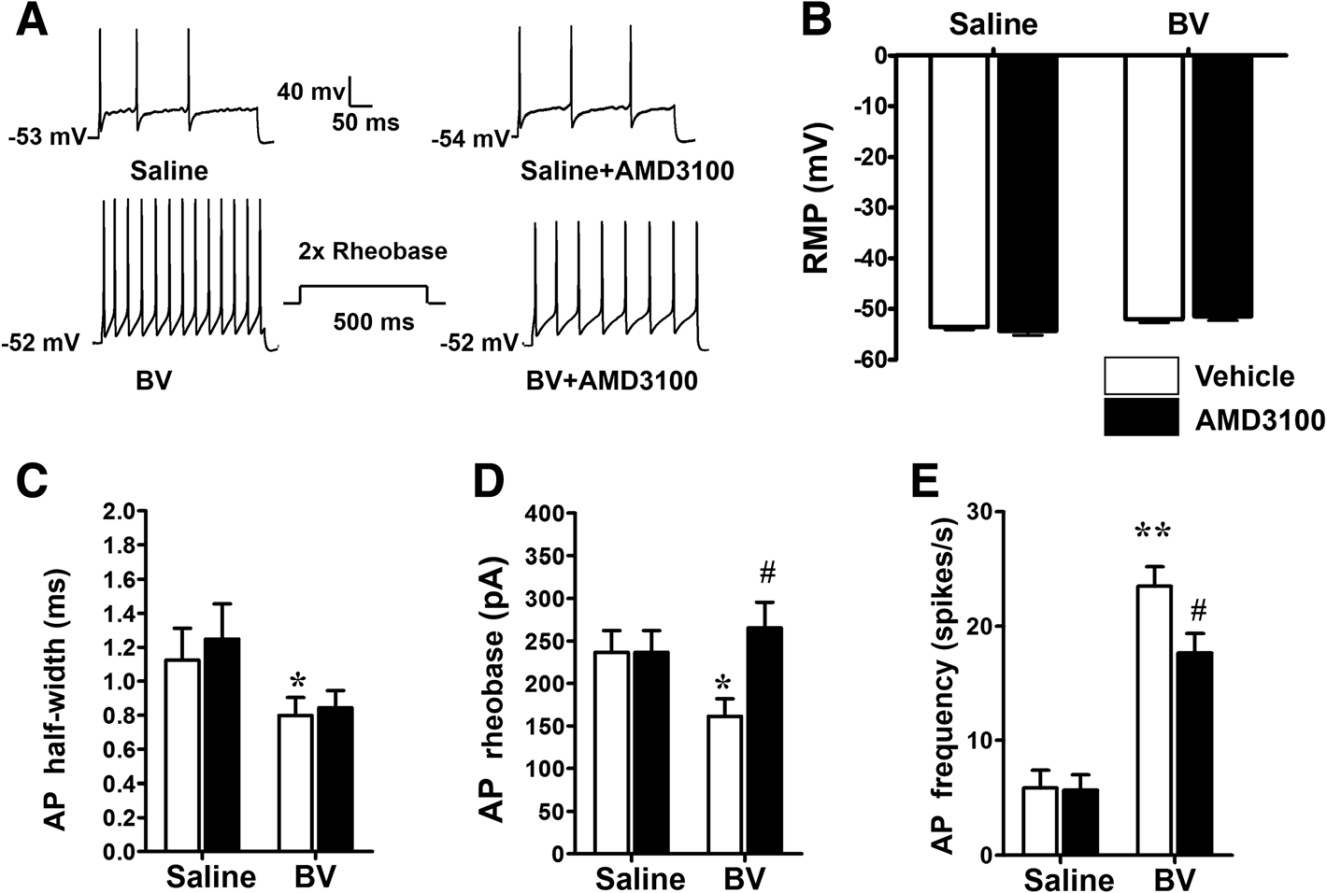
Reversal of BV-induced hyperexcitability of small- and medium-sized DRG neurons by antagonism of CXCR4. **a** Representative traces of current-evoked action potentials (AP) in the DRG neurons harvested from saline-treated and BV-treated rats. **b**–**e** Histograms exhibiting the effect of bath application of AMD3100, a selective antagonist of CXCR4, on the absolute values of RMP (**b**), AP half-width (**c**), AP rheobase (**d**), and AP frequency (**e**) in DRG neurons from saline-treated and BV-treated rats. *RMP* rest membrane potential, *AP* action potential; *n* = 8/group,*P < 0.05, **P < 0.01 BV + vehicle vs. saline + vehicle; #*P* < 0.05 BV + AMD3100 vs. saline + AMD3100

Figure 3a showed an example of current patch-clamp recording of tonic type of primary nociceptor neurons in which the firing rate was significantly increased in the BV-treated group relative to the saline control (left panel of Fig. 3a). The averaged number of APs produced in the BV-treated group was about fivefold more than the saline control group (Fig. 3e, **P < 0.01 BV vs. saline, *n* = 10 cells for each group). In the BV-treated group, the rheobase and half-width of APs were significantly reduced relative to saline control group (Fig. 3c, d *P < 0.05 BV vs. saline, *n* = 10 cells for each group); however, the resting membrane potential (RMP) was not changed between the two groups of DRG neurons (Fig. 3b). These results were also consistent with our previous report [41].

To test the role of CXCR4 in mediation of the changed excitability of DRG neurons, the effects of bath perfusion with AMD3100 (1 μM) to the DRG neurons were studied. Bath perfusion of AMD3100 could inhibit the firing rate (#*P* < 0.05 AMD3100 vs. vehicle, *n* = 10 for each group) and reverse the lowered rheobase value to the normal level (#*P* < 0.05 AMD3100 vs. vehicle, *n* = 10 for each group); however, the drug had no effect on the lowered AP half-width in the BV-treated group (Fig. 3). Bath perfusion of AMD3100 had no effect on either membrane properties or firing rate in saline control group (Fig. 3).

### Suppression of BV-induced up-regulation of Nav1.8 and phosphorylated ERK by blocking CXCR4

Based upon our present and previous data, it is reasonable to link the functions of SDF1–CXCR4 signaling with expression of Nav1.8 due to the following accumulating evidence: (1) the hyperexcitability of the tonic type of DRG neurons induced by peripheral inflammatory pain state was associated with up-regulation of Nav1.8 and Nav1.9 [28, 29, 41], (2) anti-sense down-regulation of Nav1.8 or Nav1.9 resulted in reversal of altered excitability of DRG neurons induced by peripheral inflammatory pain state [28, 29], and (3) the hyperexcitability of the tonic type of DRG neurons induced by peripheral inflammatory pain state could be blocked by antagonism against CXCR4 with AMD3100 (see in the current study). Thus, we proposed that activation of SDF1–CXCR4 signaling should be involved in regulation of Nav1.8 expression through an unknown molecular downstream route. Because it has been suggested that ERK, a subfamily of mitogen-activated protein kinase (MAPK), is a common intracellular downstream messenger of SDF1–CXCR4 signaling [42–44], examination of the roles of ERK is also reasonable.

**Fig. 4:**
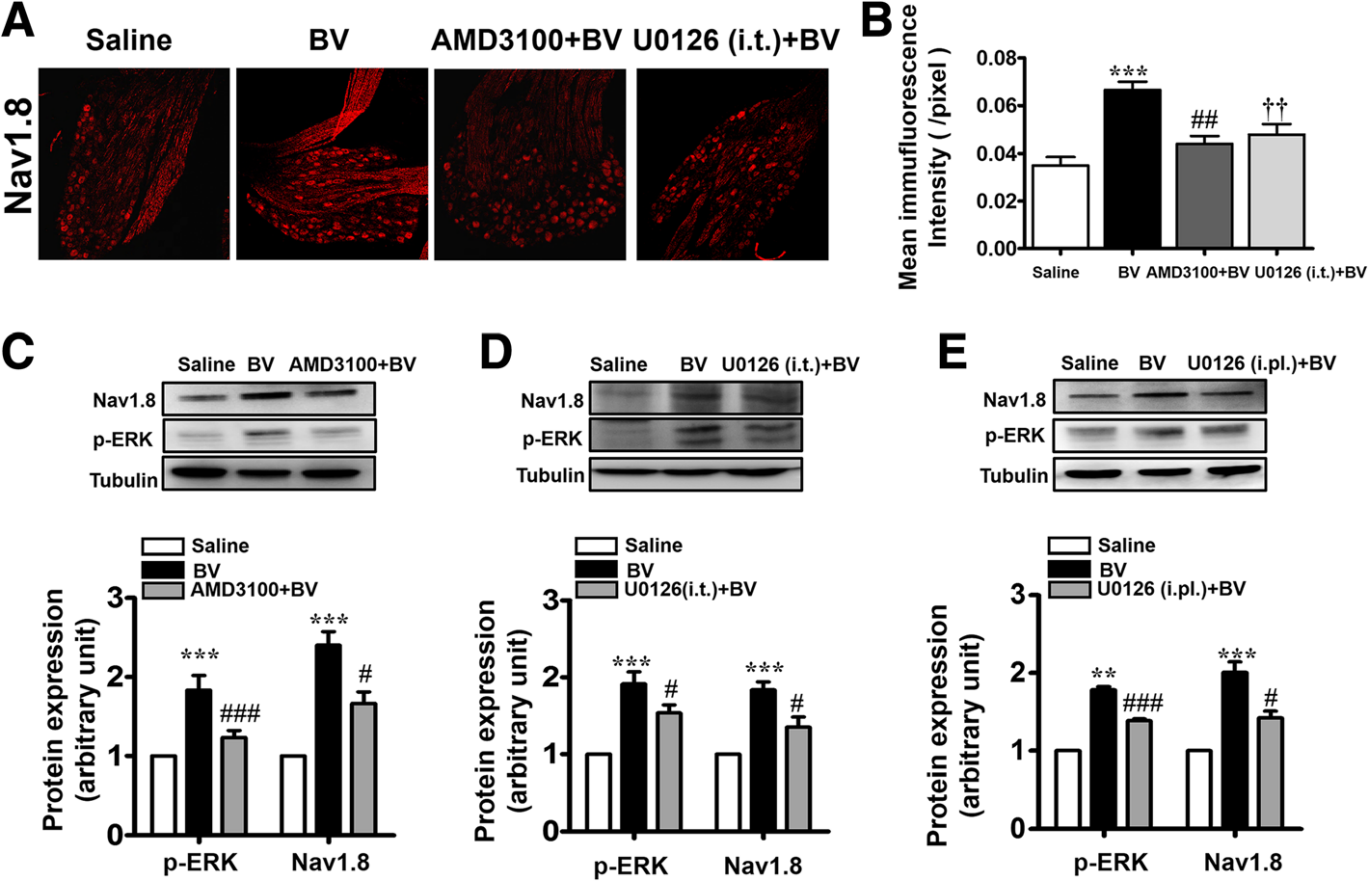
Blockade of BV-induced phosphorylation of ERK and up-regulation of Nav1.8 protein expression in the lumbar DRG by peripherally local injection of AMD3100. **a** Representative immunofluorescent photomicrographs showing expression of Nav1.8 in the lumbar DRG from saline-treated rats, BV-treated rats, and rats receiving intraplantar AMD3100 or intrathecal U0126 10 min before intraplantar BV injection. **b** Quantification of the mean immufluorescent intensity of Nav1.8 showing an increase in expression following intraplantar (i.pl.) BV injection, and a reduction in rats receiving AMD3100 or U0126 administration 10 min prior to BV (*n* = 5/group, ***P <0.001, ##*P* < 0.001, ††*P* < 0.001 vs. saline). **c** Western blot showing that AMD3100 prevented the BV-induced up-regulation of Nav1.8 and pERK activation in lumbar DRG from occurring (*n* = 3/group, ***P < 0.001 vs. saline; #*P* < 0.05, ###*P* < 0.001 vs. BV). **d** Western blot showing that intrathecal U0126 prevented the BV-induced up-regulation of Nav1.8 and pERK activation in lumbar DRG from occurring (*n* = 3/group, ***P < 0.001 vs. saline; #*P* < 0.05 vs. BV). **e** Western blot showing that intraplantar U0126 prevented the BV-induced up-regulation of Nav1.8 and pERK activation in lumbar DRG from occurring (*n* = 3/group, **P < 0.01, ***P < 0.001 vs. saline; #*P* < 0.05, ###*P* < 0.001 vs. BV)

We therefore firstly evaluated whether the BV-induced overexpression of Nav1.8 could be blocked by antagonism against CXCR4 and inhibition of activation of ERK. As shown in Fig. 4a and b, pre-treatment with i.pl. AMD3100 and i.t. U0126, respectively, could remarkably block the increased expression of Nav1.8 in the DRG neurons induced by i.pl. BV injection. Using Western blot, it was also confirmed that the BV-induced up-regulation of both Nav1.8 and phosphorylated form of ERK could be blocked by i.pl. AMD3100 as well as by i.t. U0126 (Fig. 4c and d). Moreover, as shown in Fig. 4e, pre-treatment with i.pl. U0126 could also block the increased expression of Nav1.8 in the DRG neurons induced by i.pl. BV injection.

### Relief of BV-induced persistent spontaneous nociception and primary pain hypersensitivity by peripherally local injection of AMD3100

**Fig. 5:**
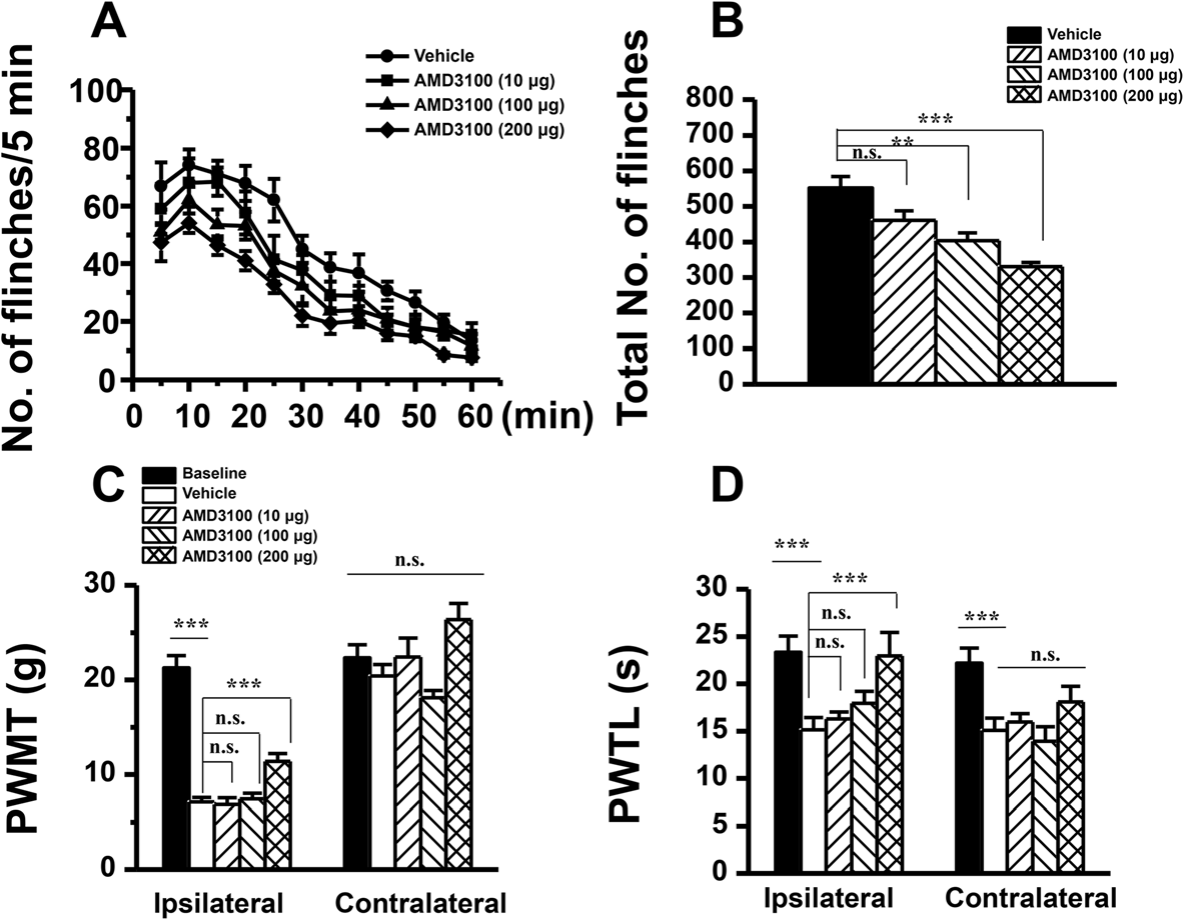
Effects of peripherally local injection of AMD3100 on BV-induced persistent spontaneous nociception and mechanical and thermal hyperalgesia. **a** Curve graphs showing the effect of AMD3100 on the persistent spontaneous nociception evaluated by recording the spontaneous paw flinches at each 5 min interval for 1 h immediately after intraplantar BV injection. **b** Column graphs showing the effect of AMD3100 on the mean total numbers of paw flinches averaged from the 1 h period intraplantar BV injection. **c**, **d** Exhibiting the effects of AMD3100 on the PWMT and PWTL measured in bilateral hindpaws of BV-treated rats. *PWMT* paw withdrawal mechanical threshold, *PWTL* paw withdrawal thermal latency; **P < 0.01, ***P < 0.001, *n* = 8–10 for each group

During the 1-h time course of the BV-induced PSN, peripherally local pre-treatment with AMD3100 (100 and 200 μg/20 μl) resulted in a dose-related suppressive effect on the development of paw-flinching reflex relative to the vehicle control, while the lowest dose (10 μg/20 μl) had no significant effect (Fig. 5a). The averaged inhibition rate produced by AMD3100 was 26.90 % for 100 μg and 40.17 % for 200 μg, respectively (Fig. 5b).

As for the effects of AMD3100 on the pain hypersensitivity, only the highest dose of AMD3100 (200 μg/20 μl) could significantly prevent the development of both primary heat and mechanical hypersensitivity but with the mirror-image heat hypersensitivity being unaffected (Fig. 5c, d). The same treatment of AMD3100 even at the highest dose used in the present study had no influence upon the basal pain sensitivity to either heat or mechanical stimuli (data not shown).

### Involvement of ERK signaling and Nav1.8 in the development and maintenance of BV-induced persistent spontaneous nociception and primary pain hypersensitivity

**Fig. 6:**
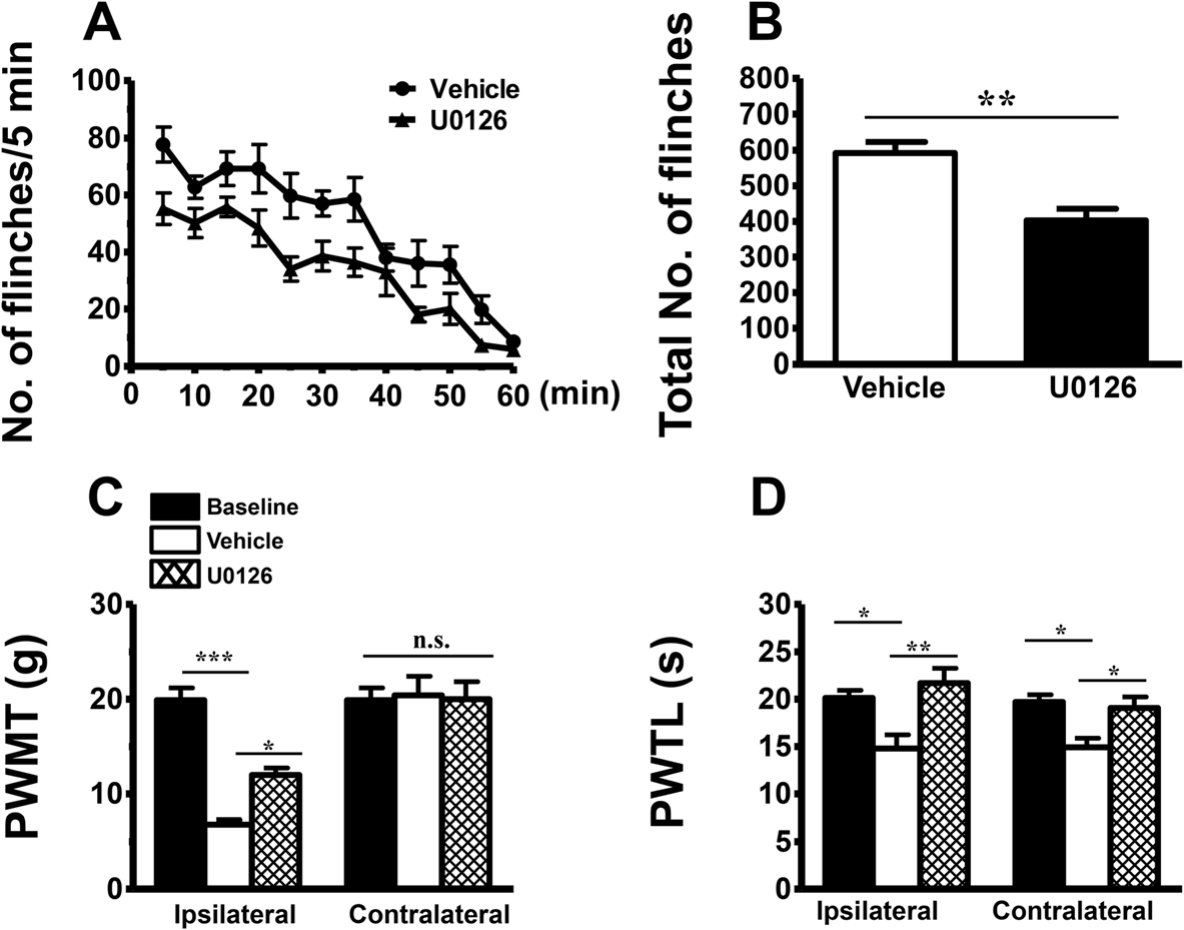
Effects of intrathecal administration of an ERK inhibitor, U0126, on BV-induced persistent spontaneous nociception and mechanical and thermal hyperalgesia. **a** Curve graphs showing the effect of U0126 on the persistent spontaneous nociception evaluated by recording the spontaneous paw flinches at each 5 min interval for 1 h immediately after intraplantar BV injection. **b** Column graphs showing the effect of U0126 on the mean total numbers of paw flinches averaged from the 1 h period intraplantar BV injection. **c**, **d** Exhibiting the effects of U0126 on the PWMT and PWTL measured in bilateral hindpaws of BV-treated rats. *PWMT* paw withdrawal mechanical threshold, *PWTL* paw withdrawal thermal latency; *P < 0.05, **P < 0.01, ***P < 0.001, *n* = 6 for each group

**Fig. 7:**
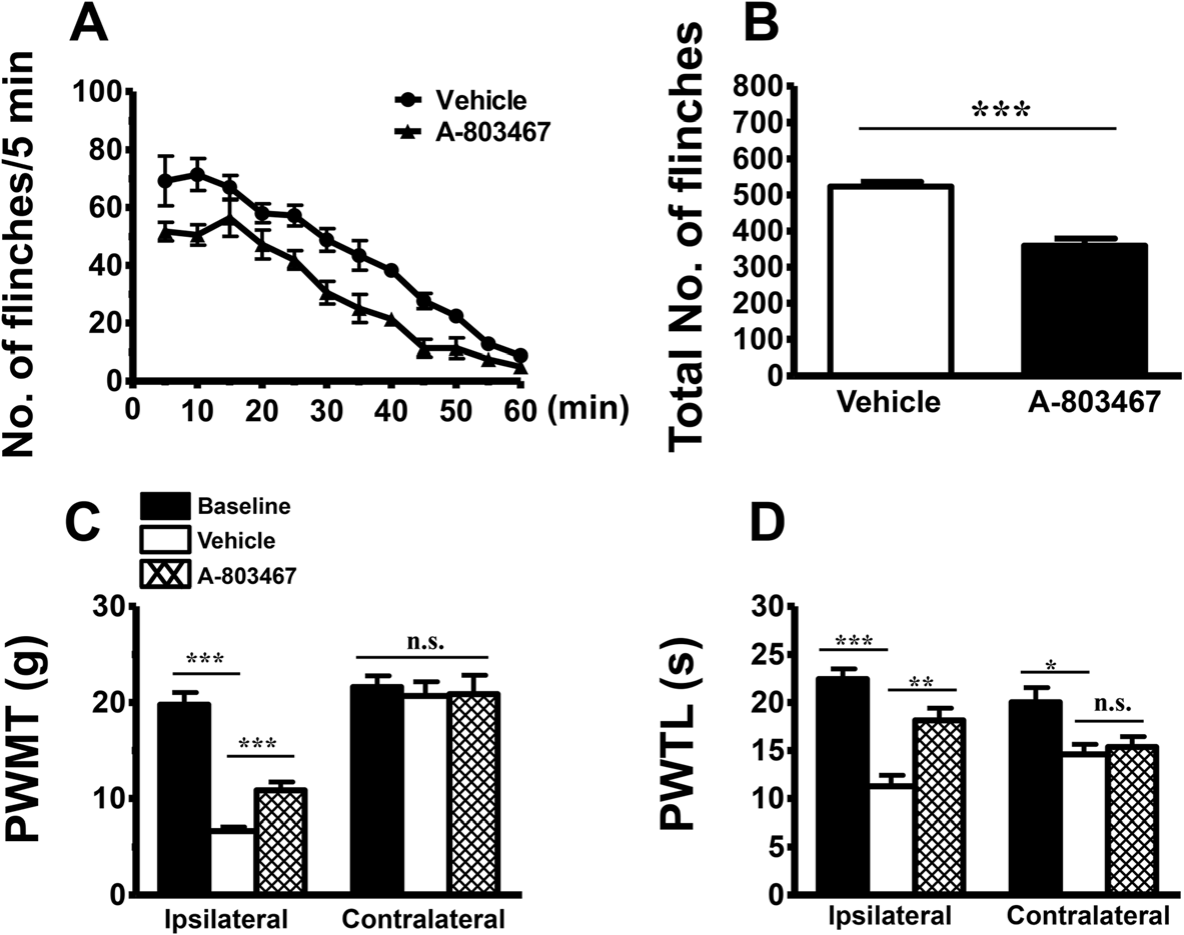
Effects of intraplanter injection of a Nav1.8 blocker, A-803467, on BV-induced persistent spontaneous nociception and mechanical and thermal hyperialgesia. **a** Curve graphs showing the effect of A-803467 on the persistent spontaneous nociception evaluated by recording the spontaneous paw flinches at each 5 min interval for 1 h immediately after intraplantar BV injection. **b** Column graphs showing the effect of A-803467 on the mean total numbers of paw flinches averaged from the 1 h period intraplantar BV injection. **c**, **d** Exhibiting the effects of A-803467 on the PWMT and PWTL measured in bilateral hindpaws of BV-treated rats. *PWMT* paw withdrawal mechanical threshold, *PWTL* paw withdrawal thermal latency; *P < 0.05, **P < 0.01, ***P < 0.001, *n* = 6 for each group

As shown in Figs. 6a and 7a, i.pl. BV injection produced a rapid-onset, long-term spontaneous flinching reflex which could last for 1–2 h. Comparing with vehicle-treated control group, i.t. pre-treatment with U0126 (10 μg/10 μl, 10 min before) significantly prevented the development of persistent spontaneous nociception (Fig. 6a, b). Likewise, ipsilateral i.pl. treatment with A-803467 (500 μg/50 μl) 10 min before BV injection also prevented the occurrence of persistent flinching reflex (Fig. 7a, b).

In comparison with vehicle group, pre-treatment with i.t. U0126 (10 μg/10 μl) prevented the development of primary mechanical and heat hypersensitivity identified in the hindpaws ipsilateral to BV injection and the mirror-image heat hypersensitivity in the contralateral hindpaws (Fig. 6c, d). Figure 7c and d showed that the established primary mechanical and heat hypersensitivity could be reversed by i.pl. treatment with A-803467 (500 μg/50 μl) 90 min post-BV injection, while the mirror-image heat hypersensitivity remained unaltered.

## Discussion

In the present study, we demonstrated that the SDF1–CXCR4 signaling contributes to the hyperexcitability of tonic type of the primary nociceptor cells through up-regulation of Nav1.8 via regulating ERK pathway. Blocking the SDF1–CXCR4 signaling as well as ERK activation and Nav1.8 activity can suppress both the persistent spontaneous nociception and primary pain hypersensitivity through down-regulation of both ERK and Nav1.8. Taken together, it is concluded that SDF1–CXCR4 signaling contributes to persistent pain and hyperalgesia (and allodynia) via regulating the excitability of primary nociceptive neurons maintained by ERK-dependent Nav1.8 up-regulation.

### Maintenance of primary nociceptor neuronal hyperexcitability by SDF1–CXCR4 signaling under peripheral inflammatory pain state

In the present study, it is interesting to note that antagonism of CXCR4 by bath perfusion of DRG with AMD3100 results in significant inhibition of the BV-enhanced neuronal firing rate by reversing the lowered rheobase value to the normal level. This result suggests a maintaining role of SDF1–CXCR4 signaling in the primary nociceptor hyperexcitability at the neuronal cell body level caused by peripheral inflammatory pain state. The primary nociceptor neuronal cell body hyperexcitability caused by the BV-induced peripheral inflammatory pain state has already been demonstrated in our previous reports [28, 29, 40, 41]. Regarding this, one question may arise to be asked: which cell types are possible sources of SDF1 and CXCR4 in the DRG? To answer this question, we further demonstrated that, by double immunofluorescent staining and/or Western blotting, SDF1 was exclusively up-regulated in GFAP-positive non-neuronal SGCs of the DRG following i.pl. BV injection, while CXCR4 was mainly co-localized with IB4, SP, and TRPV1, specific biomarkers of primary nociceptor neurons in the DRG. This at least suggests that the non-neuronal SGCs can be activated by peripheral inflammatory pain state that serve as a source of SDF1 release, allowing its binding to CXCR4 be over expressed in the primary nociceptor neuronal cell body caused by persistent firing initiated in the peripheral terminals [33, 45–47]. The mechanism of the activation of non-neuronal SGCs may be due to the fractalkines released from DRG neurons which has been demonstrated by Souza and colleagues in i.pl. carrageenan injection-induced inflammatory pain model [48]. It has been demonstrated that, under physiological state, both primary afferent neurons and non-neuronal SGCs in the DRG constitutively contain SDF1 that is maintained at a very low level [9]. However, the source of SDF1 under pathological level may vary depending upon different conditions. In antiretroviral toxic neuropathy model, Bhangoo and colleagues have observed up-regulation of SDF1 and CXCR4 mRNA at 7 and 14 days after administration of antiretroviral drug 2, 3-dideoxycytidine, and the enhanced expression of SDF1 was mostly observed in the DRG non-neuronal cells [49]. Similarly, Dubovy and colleagues have also demonstrated that the non-neuronal SGCs were the source of SDF1 by showing co-localization of SDF1 and glutamine synthetase in the DRG of animals with neuropathic pain induced by CCI [15]. Based upon the above lines of evidence, it is highly suggested that the inducible release of SDF1 from non-neuronal SGCs by peripheral inflammatory and neuropathic pain conditions should result in development of intraganglionar inflammatory microenvironment, by which long-term hyperexcitability of primary nociceptor neurons can be maintained. As lines of supporting evidence, it has been demonstrated that the SGCs in the DRG when they had been activated by i.pl. carrageenan injection could produce some pro-inflammatory mediators (TNFα, IL-1β, and prostanoids) that can directly excite the primary nociceptive neurons [48]. Moreover, SDF1 has been shown to significantly increase intracellular calcium concentrations in the isolated DRG neurons under several persistent pain conditions [49–51]. It has also been demonstrated that exogenous SDF1 could lower the threshold for action potential generation and depolarize nociceptive DRG neurons in cultured DRG neurons [21]. However, sensory neurons of the DRG may also be the source of SDF1 since increase in SDF1 mRNA or protein have been observed in DRG neurons of transgenic mice with high-fat diet-induced type II diabetic neuropathy [50] and animals receiving repeated morphine treatment [51]. Besides SDF1, other chemokines are also likely to be involved in primary sensory neuronal hyperexcitability, because up-regulation of CCL2, CCL3, and CXCL1 by pain and direct activation of DRG neurons by them have been seen in several previous reports [6, 30, 31, 52, 53].

As for the source of CXCR4, a selective cognate receptor of SDF1, only primary sensory neurons have been targeted. Consistent with some previous reports [9, 16, 21], in our present study, CXCR4 was constitutively present but overexpressed under peripheral inflammatory pain state in both non-peptidergic (IB4-positive) and peptidergic (SP-positive) primary nociceptor neurons. It is also co-localized with TRPV1, a thermonociceptor of primary sensory afferent. However, the overexpression of CXCR4 induced by intraplantar BV injection could be significantly prevented by pre-treatment with i.pl. AMD3100, a selective CXCR4 inhibitor, suggesting that the BV-induced overexpression of CXCR4 in the DRG cells may be partially mediated by peripheral SDF1–CXCR4 signaling in the skin which was trafficking from neuronal soma to the peripheral terminals. In the case of SDF1–CXCR4 signaling pathway, Reaux-Le and colleagues have found that CXCR4 receptor was constitutively present in both peptidergic (CGRP positive) and non-peptidergic (IB4 positive) DRG neuronal soma in rats [9]. They have also demonstrated that CXCR4 can be localized in pre-synaptic components of both type I and type II glomeruli in the spinal dorsa horn under electron microscope, indicating that CXCR4 could be axonally transported to both peripheral and central terminals of the primary afferent neurons in DRG and exert its functions [9]. Although we do not detect the protein level of CXCR4 in the skin directly in the current study, Reaux-Le and colleagues’ results can lend support to the rationale of peripheral administration of AMD3100 in our current study because CXCR4 immunoreactivities co-expressed in the CGRP-positive fibers have been shown to be present in the glabrous skin as well as in the DRG cells and project to the dermis [9]. The functional nature of this increased CXCR4 receptor expression was identified by our behavioral pharmacology assays in which pre-treatment with i.pl. AMD3100 significantly prevented the development of the BV-induced persistent spontaneous pain-related behaviors and pain hypersensitivity. Moreover, bath perfusion of medium- and small-sized DRG neurons with AMD3100 also significantly reduced BV-induced tonic discharges by restoration of rheobase value to normal level, suggesting a maintaining role of SDF1–CXCR4 signaling in the primary nociceptor hyperexcitability. Since it is known that the decrease in rheobase value may reflect changes in persistent Na + conductance [54, 55], the roles of TTX-resistant VGSC α subunits Nav1.8 and/or Nav1.9, which are selectively expressed in the primary nociceptor neurons, should be further investigated. As aforementioned, we have already demonstrated that Nav1.8 and Nav1.9 could be up-regulated in the small and medium-sized DRG cells by i.pl. melittin, the major toxin of whole bee venom, or CFA injection, resulting in increased persistent current mediated by Nav1.8 and Nav1.9 and enhanced firing rate of tonic, but not phasic, type of primary nociceptor neurons with lowered rheobase value [28, 29, 41]. Down-regulation of Nav1.8 and Nav1.9 by anti-sense oligodeoxynucleotide, respectively, resulted in restoration of Nav1.8 and Nav1.9 current density and rheobase value, leading to reduction of hyperexcitability of primary nociceptor neurons and relief of persistent nociception and pain hypersensitivity induced by i.pl. injection of melittin or CFA [28, 29]. In the present, our behavioral data also demonstrated that peripheral pre-treatment with A-803467, a selective Nav1.8 blocker, could inhibit the persistent nociception and reverse the primary pain hypersensitivity induced by i.pl. BV injection, indicating that increased expression of Nav1.8 in the DRG induced by i.pl. BV injection contributed to the development and maintenance of the BV-induced pain-related behaviors. Taken together, we proposed that there must be a functional link between SDF1–CXCR4 signaling and expression of Nav1.8 or Nav1.9 that maintains primary nociceptor neuronal hyperexcitability under peripheral inflammatory pain state.

### Involvement of SDF1–CXCR4 signaling in the up-regulation of Nav1.8 sodium channels in DRG neurons via ERK-dependent pathway

In the present study, we further demonstrated that peripheral antagonism against CXCR4 pharmacologically with AMD3100 10 min prior to i.pl. BV could prevent up-regulation of Nav1.8 sodium channel from occurrence in the DRG. The phosphorylation of ERK (pERK), an isoform of MAPK, was also significantly suppressed in the DRG by pre-treatment of AMD3100 at the i.pl. BV injection site. The phosphorylation of ERK has been shown to be critical for up-regulation of Nav1.8 [56]. CXCR4 activation has also been shown to induce multiple intracellular signal transduction pathways, including ERK signaling pathway [42–44]. Both intrathecal and intraplantar pre-treatment of U0126, an ERK inhibitor, could inhibit the level of pERK and expression of Nav1.8 in the DRG. Furthermore, consistent with our previous findings that peripheral ERK contributes to the persistent pain induced by melittin, we here found that the BV-induced persistent nociception and primary mechanical and thermal hypersensitivity could be inhibited by pre-treatment with i.t. U0126 and i.pl. A-803467, respectively. These results suggest that SDF1–CXCR4 signaling should be involved in up-regulation of Nav1.8 through an ERK-dependent pathway in the primary nociceptor neurons following i.pl. BV injection, which contributes to hyperexcitability of the primary nociceptor neurons that mediate maintenance of both persistent spontaneous nociception and primary pain hypersensitivity. In support, up-regulation of Nav1.8 sodium channel by chemokine CCL2 has been seen in acutely dissociated and cultured DRG neurons from naïve rats that are likely to be mediated by PKC-NFκB and Gβγ-dependent mechanisms [30, 57, 58]. The expression level of Nav1.8 has also been demonstrated to be up-regulated by TNFα or down-regulated by interleukin-10 (IL-10) in rat DRG neurons [26, 59]. However, unlike the release of SDF1 from the SGCs, the source of chemokine CCL2 and pro-inflammatory mediators TNFα and IL-10 were thought to be released through paracrine or autocrine from DRG neurons that possess their respective receptors [26, 30, 57–59]. Taken ours and previous results together, it seems that there are at least two regulating patterns to be involved in up-regulation of Nav1.8 in the DRG under peripheral inflammatory and neuropathic pain conditions: one is likely to be initiated by neuronal activity-dependent autocrine–autoreceptor pattern mediated by TNFα-TNFR and/or CCL2-CCR2 signaling pathways within the primary nociceptor neurons [26, 30, 57–59], while the other is likely to be initiated by SGC-neuronal pattern mediated by SDF1–CXCR4 signaling pathway. We propose that the autocrine–autoreceptor pattern should be involved in the early induction process, while the satellite glial-neuronal pattern in the late maintaining process of both peripheral inflammatory and neuropathic pain conditions. As a line of supporting evidence, pro-inflammatory mediator TNFα has been shown to be only involved in the induction of primary nociceptor hyperexcitability but not involved in the maintaining process [26, 59]. Contrarily, the SGC-neuronal pattern mediated by SDF1–CXCR4 signaling pathway was shown, in the current study, to be involved in the maintaining process because the well-established BV-induced primary nociceptor neuronal firing could be suppressed by bath perfusion of the DRG with AMD3100, thus providing with a novel molecular target for treatment of clinical pain.

Nav1.8 is a sensory neuron-specific channel which acts as a major contributor to the upstroke of action potentials and supports repetitive firing in response to depolarizing input, and is preferentially expressed in nociceptive DRG and trigeminal ganglion neurons [24, 60–62]. By using diphtheria toxin to kill all Nav1.8-positive sensory neurons, mechanical, cold, and inflammatory pain have been demonstrated to be ameliorated [63]. Nav1.8-null mutant mice have been shown to have weak responses to noxious cold and mechanical stimulation [64]. Moreover, intraperitoneal administration of A-803467, a potent Nav1.8 sodium channel blocker, has been shown to produce significant anti-nociception in inflammatory pain models induced by CFA and capsaicin [65]. Collectively, these findings suggest that Nav1.8 may be a good target for the development of novel analgesics for treatment of inflammatory pain hypersensitivity [66]. However, the side effects, which were produced due to the high degree of structural homology within the VGSC family, and the poor bioavailability of the existing VGSC blockers limit their clinical use [66–68]. Based upon our present result, targeting the SDF1–CXCR4 signaling between SGC-neuronal link might be a good option for treatment of chronic pain. Because the SDF1–CXCR4 signaling may also regulate other various downstream ion channels such as other subtypes of VGSC, voltage-gated calcium channels, and the family of TRP (TRPV, TRPA, TRPM, TRPC) channels, further intensive studies of the regulating functions of SDF1–CXCR4 signaling at the DRG neuronal cell body level may shed new light on the treatment of chronic inflammatory and neuropathic pain conditions.

## Conclusions

In summary, our present data demonstrated that, under the peripheral inflammatory pain condition, SDF1–CXCR4 signaling between non-neuronal SGCs and primary nociceptor neurons is dramatically enlarged through up-regulation of both substances in the DRG which acts as a mediator for the intraganglionar neuroinflammatory microenvironment and induces ERK-dependent up-regulation of Nav1.8 that contributes to the maintaining process of primary nociceptor neuronal hyperexcitability that is required for maintenance of persistent spontaneous pain and hypersensitivity.
